# Comparative outcome analysis of home-initiated non-medical interventions among toddlers with orally ingested substances

**DOI:** 10.1186/s13052-015-0170-2

**Published:** 2015-09-15

**Authors:** Menyfah Q Alanazi, Majed I Al-Jeraisy, Mahmoud Salam

**Affiliations:** Drug Policy and Economic Center, Riyadh, Saudi Arabia; King Abdullah International Medical Research Center (KAIMRC), Riyadh, Saudi Arabia; King Saud bin Abdulaziz University for Health Sciences (KSAU-HS), Riyadh, Saudi Arabia

## Abstract

**Background:**

Poison management guidelines recommend contacting or visiting poison centers directly after exposure. However, some parents initiated non-medical interventions on their children before visiting these centers. Aim was to evaluate the clinical and hospital outcomes of such practices among toddlers with orally ingested medication or chemical substances at a tertiary care facility.

**Methods:**

Retrospective cohort, based on four-arm outcome analysis. Exposures were gender, age, body mass index, arrival time to facility (hours) presented in Median [Interquartile range]. Clinical outcomes were vital signs, physical examination, diagnostic tests; Hospital outcomes were in-hospital admission, length of hospital stay (hours) presented in Median [Interquartile range], hospital cost ($US). Bivariate analysis (nonparametric tests), binary logistic/linear regression were conducted. Significance at p < 0.05.

**Results:**

Between 2009–2011, 165 (all previously healthy) toddlers were (Males = 58 %, females = 42 %) and had normal weights in 70 %. Witnessed incidents were in 85 %. Two control groups [Medication (control) = 72, Chemical (control) = 48] directly visited the facility after incident, while two intervention groups [Medication (intervention) = 27, Chemical (intervention) = 18] received orally administered water, salt/sugar solutes, milk/yogurt, lemon juice and/or manually induced vomiting before the visit. Abnormal clinical outcomes in total were in vital signs = 15 %, physical examination = 42 % and diagnostic tests = 26 %; hospital outcomes were admission = 16 %, length of stay range (2 hours–7.5 days), cost range (667–11,500). Bivariate analysis: Length of stay in Medication (intervention) = 9[5.4–12.0] hours significantly higher than Medication (control) = 5[2.7–7.5] hours, p = 0.003; abnormal physical examination in Chemical (intervention) = 77.8 % significantly higher than Chemical (control) = 37.5 %, p = 0.004. In regression: intervention significantly increased length of stay (t = 0.213, adj. P = 0.035); lower weight toddlers were at higher risk of admission (Beta = -0.51, adj. P = 0.018); delayed arrival time significantly increased abnormal physical examination (Beta = 0.29, adj. P = 0.003). No significant control/intervention group differences regarding abnormal vital signs (adj. P = 0.148), physical examination (adj. P = 0.781), diagnostic tests (adj. P = 0.285), admission (adj. P = 0.499), and cost (adj. P = 0.102).

**Conclusion:**

Home-initiated non-medical interventions didn't improve the clinical and hospital outcomes. It has delayed the arrival time to emergency department, which added the risk of encountering abnormal physical examination, and in return increased the average length of hospital stay.

## Background

One of the most unfortunate events toddlers may encounter during their early years of curiosity and experimentation is substance poisoning [1, 2]. Poison control centers in the United States received more than 2.4 million poison case reports in 2003, of which 45.7% were aged =< 3 years [3]. One of the key organizations that focuses its efforts on poison prevention and management initiatives is the American Academy of Pediatrics (AAP) [4]. Pooling of millions of poison reports among children has generated solid evidence based recommendations and management guidelines which dramatically decreased such unfortunate events over the years [4].

Hospital poison management is based on an appropriate supportive and/or toxic-specific treatment [5–9]. At homes, IPECAC had been recommended as a safe emetic between 1965 and 2003 [10]. However, the American Association of Poison Control Centers (AAPCC) in its 2011 report stated that IPECAC altered the child’s tolerance to orally ingest hospital poison treatments and should no longer be used at homes [4, 5, 11]. Activated charcoal usage dates back further as a traditional gastric decontaminant [12], yet its routine usage is discouraged, especially after one hour of substance ingestion [6, 13]. In addition, some guidelines recommended dilution by drinking 100 to 200 mL of water, but only for chemical substance ingestions [4].

Poison center experts generally discourage any sort of home-initiated non-medical intervention and advise parents to notify poison centers or visit the emergency department (ED) for professional management [14, 15]. However, it was reported in a tertiary care facility that some parents took the initiative of performing unconventional poison management for their children prior the ED visit, such as oral administration of water, lemon juice, milk, yogurt, sugar, salt and/or manually induced vomiting.

Food-drug interactions are known to increase, neutralize or cease the desired effect of some medications [16]. This may be limited to pharmacological doses, but not toxicological doses of medications. Manually induced vomiting exists more frequently among adults with binge eating disorders (self-provoked) [17]. To our knowledge this existing home-initiated nonmedical poison management was not attended to in previous studies, especially among the high risk group of toddlers [2, 3, 13, 18]. This practice may or may not improve the clinical and hospital outcomes.

Aim was to evaluate the outcomes of home-initiated non-medical interventions among toddlers complaining of orally ingested substances admitted to an ED of a tertiary care facility, central Saudi Arabia. This was fulfilled by: 1. Screening for toddlers with acute poisoning (medication or chemical), 2. Assessing for a number of exposure variables, 3. Identifying the control and the intervention groups, 4. Evaluating and comparing the clinical and hospital outcomes.

## Patients and methods

### Study design

This is a retrospective cohort, based on a four-arm comparative outcome analysis.

### Study area/setting

King Abdulaziz Medical City (KAMC) is a distinguished Joint Commission International (JCI) accredited tertiary health care facility established in 1983 and under the umbrella of the Ministry of National Guard Health Affairs (MNG-HA). With a total bed capacity of 1,200 beds, the ED at KAMC has 125 beds allocated for adult/pediatric wards of various care levels. A team of more than 110 emergency specialized consultants, associate consultants, assistant consultants, staff physicians, fellows and residents provide services to an estimate of 36,000 ED admissions annually [19].

KAMC has a certified poison center under the name of clinical toxicology department which is enlisted - in addition to other local poison centers - under the National Drug & Poison Information Center (NDPIC). NDPIC reports to the Saudi Food and Drug Authority (SFDA). Similar to any poison center, it has oncall toxicologists who respond to any public or health care professional queries regarding any incident of substance ingestion and provides emergency guidance [20, 21].

### Study subjects and sampling technique

**Fig. 1:**
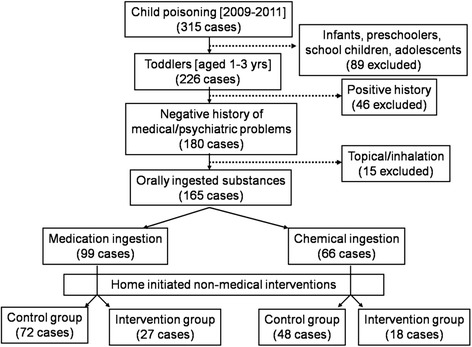
Inclusion/exclusion criteria and distribution of cases between control and intervention groups

By convenience, all children admitted to the ED of KAMC complaining of acute poisoning (medication and/or chemical substance) between 2009 and 2011 were screened based on a preset inclusion/exclusion criteria (Fig. 1). Oral ingestion cases were only enrolled since home-initiated non-medical interventions were intended for oral exposure route. Any case with a previous health condition, such as asthma, was excluded to control for any potential confounder that may result in a more complicated or deteriorated clinical outcome. Cases of intentional over-dosage or suspected domestic violence were excluded too.

At the time of ingestion, none of the toddlers’ parents notified KAMC by phone prior their visit to ED. The decision of whether to directly visit ED (control group) or initiate a non-medical intervention at home then visit ED (intervention group) was based on the parent’s sole decision. Therefore, accurate evaluation of the outcomes was based on four group comparisons: two medication and chemical ingestion control groups (Med^control^ & Chem^control^) matched and compared to two intervention groups (Med^interv^ & Chem^interv^).

### Data collection

Data collection team constituted of various full-time health care professionals at KAMC. This team was lead and managed by two certified clinical research coordinators from King Abdullah International Medical Research Center (KAIMRC). Team members were trained by study investigators on how to screen for eligible study subjects, obtain informed consent from one of the parents, conduct a face-to-face interview and follow-up on the clinical and hospital outcomes. Follow-up was conducted by tracing study subjects using a secure access to the medical records stored in an electronic database called (Quadra-Med). An agreement between the ED and study investigators was made to instantly report to the data collection team any child arriving with a suspected poisoning. The investigators closely monitored the enrollment of study subjects. Validation of data was done by verifying the results in the medical records and contacting parents by phone. Phone calls after the toddler has been released from the hospital were very important as questioning the anxious and stressed parents during the ED visit often leads to an inaccurate history or description of the intervention [10, 21].

Some of the components in the data collection tool were sourced from the standardized local Ministry of Health (MOH) and DPIC reporting forms of drug over dosage or chemical poisoning. It is composed of:

1. Informed consent: name of the toddler and parents, medical record number (MRN), date/time, contact information and signatures all reported by parents.
2. Toddler characteristics: age, sex, previous medical/psychiatric history and body parameters (height and weight), body mass index (BMI) calculated only for 2–3 year old toddlers, plotted on sex-specific growth charts [22], and classified as under weight (<5^th^ percentile), healthy weight (5–85^th^ percentile), over weight (86–94^th^ percentile) and obese (≥95^th^ percentile). These characteristics were obtained during triage by health care providers at ED and members of data collection team.
3. Incident characteristics: substance type, exposure route, time of incident, witnessed or not, poison center informed or not, arrival time to ED visit (hours), and any home-initiated non medical intervention provided. These were reported by parents.
4. Outcome characteristics: Clinical outcomes included vital signs (V.S.), physical examination (P.E.) which is signs and symptoms, diagnostic tests (Diag) including lab tests, radiology and others. Abnormal findings were based on officially documented medical diagnosis, laboratory results and nursing notes on Quadra-med. Hospital outcomes included status post ED (recovered and released or in-hospital admission), length of stay (LOS) in hours, hospital bill (Cost) in US dollars. These were obtained from patient medical services and finance.

### Ethical considerations

All data collection team preserved the confidentially of the patients’ information as part of their job requirements. MRNs and contact information of enrolled subjects were recorded to follow-up on the outcomes and validate the data collected. Signed informed consents were stapled to the data collection tool and preserved in sealed envelopes upon their completion. The study investigator had no influence on which group of toddlers to receive the home-initiated non-medical intervention. Parents didn’t notify the KAMC poison center prior the conduction of such practice. The data in this study was sourced out from a broad-scale approved proposal which describes the general pattern of poisoning cases, approved by the Institution Review Board of the Ministry of National Guard Health Affairs (MNG-HA), Riyadh, Saudi Arabia (RR08/019).

### Data management and analysis

SPSS statistical software (Version 22; SPSS Inc., Chicago, IL, USA) was used for data entry and analysis. Bi-variate analysis using Pearson’s chi-square test was used for categorical data such as sex, BMI percentile category and some outcomes. Fisher’s exact test was adopted when the cells within contingency tables were of smaller frequencies. Testing for normal distribution using Kolmogorov-Smirnov was significant (*p* < 0.001), indicating a non normal distribution of the outcome continuous variables. These were presented by median (M), 25–75^th^ percentile inter-quartile range [IQR] and tested by Mann-Whitney test. Binary logistic regression and linear regression were constructed to identify the significant outcomes of home-initiated non-medical interventions; adjusted *P* value (adj. P), adjusted relative risk (adj. RR), and the 95 % confidence interval of adj. RR (CI). Significance level set at *P*-value < 0.05.

## Results

From the 165 toddlers (1–3 years) who meet the inclusion criteria, 99 (60 %) ingested various types of Med. products while 66 (40 %) ingested Chem. products. Male toddlers were at higher risk in both Med. (56.6 %) and Chem. (60.6%) groups, with no significant gender difference (*p* = 0.606). BMI calculated for 2–3 year old toddlers showed that the majority had normal weight 70.2 %, while underweight were 4.4 %, and overweight to obese 25.4%, with no significant difference between Med. and Chem. groups, *p* = 0.528. All toddlers enrolled had no previous history of medical or psychiatric problems.

In Med. group, the most common orally ingested substances were antipyretics & analgesics (*n* = 25), cardiac drugs (*n* = 10), and more than one type of medication (*n* = 13). In Chem. group, 21 toddlers ingested sodium hydroxide (household product) while 14 ingested kerosene (petroleum product). Other Med. and Chem. ingested products are enlisted in Table 1. Almost 85 % of substance ingestion incidents were witnessed by one of the parents. Besides the fact that none of the parents‛ toddlers notified the poison center after the incident, 27 % decided to initiate a non-medical intervention, then visit the ED. Mutually exclusive interventions included forcing the toddler to drink plain water (*n* = 17), lemon juice (*n* = 5), milk (*n* = 10), yogurt (*n* = 2) and salt/sugar solutes (*n* = 1). In addition, manually induced vomiting with or without fluid administration was observed (*n* = 26). The time between the incident and arrival to ED ranged between 0.3 and 3.4 hours.

**Table 1:** Frequency list of orally ingested substances

Medication substance	*n*	Chemical substance	*n*
Antipyretics/analgesics	25	Hydrogen/Ammonium hydroxide	5
Antidepressants	2	Chloroxylenol	2
Psychotics	3	Bleach	2
Neurological	6	Organophosphate	7
Hormone analogue	6	Paint thinner	3
Gastrointestinal	4	Rodenticide	2
Antibiotics	4	Alcohol based chemical	4
Creams	3	Alkaline based chemical	1
Vitamins/minerals	7	Surfactant	2
Antihistamines	9	Petroleum product	14
Contraceptives	6	Sodium hydroxide	21
Hypoglycemic	1	Natural dye	1
Cardiac drugs	10	Caustic soda	1
Multiple drugs	13	Unidentified chemical	1
Total	99	Total	66

Note: *n* number of cases

Abnormal clinical outcomes observed in all investigated toddlers were in V.S. (15 %), P.E. (42 %), and Diag tests (26 %). Gastrointestinal disturbances (*n* = 49) and hyperglycemia (*n* = 24) were the most common abnormal P.E. and Diag tests respectively. Other abnormal P.E. and Diag test findings are stated in Table 2. In addition, hospital outcomes were admission rate(16%), LOS ranged from 1 hour to 7.5 days, and cost ranged from $666 to $11,500.)

**Table 2:** List of mutually exclusive abnormal physical examination and diagnostic test findings

	Abnormal findings	Medication group(n)	Chemical group(n)
Physical Examination			
Respiratory	- Coughing, Bronchospasms	1	12
Cardiac	- Tachycardia	7	2
Gastrointestinal	- Abdominal pain, Nausea, Vomiting, Diarrhea	29	20
Dermatological	- Rash, Dermal burn, Flushing, Pale, Lip/tongue swell	6	9
Ocular problems	- Miosis, Irritation, Blurred vision	2	2
Neurological	- Altered consciousness, Ataxia, Seizure, Lethargy, Confusion, Hyperactivity	20	11
Urinary	- Retention	0	1
Diagnostic tests			
	- Hyperglycemia	9	15
	- Electrolyte imbalances	3	1
	- Abnormal ABGs	2	1
	- Abnormal Renal profile	2	0
	- Abnormal liver function test	2	2
	- High Substance level in plasma	6	0
	- Abnormal cardiac rhythm	2	0
	- Abnormal chest X-ray	1	2

Bivariate analysis of the clinical and hospital outcomes between the compared groups Med^control^ & Med^interv^; Chem^control^ & Chem^interv^ was conducted, Table 3. Initial significant outcomes within the Med groups were observed in the LOS, longer in the Med^interv^ group M = 9 [5.4–12.0] hours, compared to Med^control^ group M = 5 [2.7–7.5] hours, *p* = 0.003. On the other hand, Chem^interv^ group had significantly higher incidence of abnormal P.E. 77.8 % compared to Chem^control^ 37.5 %, *p* = 0.004. No significant differences were observed regarding other clinical and hospital outcomes.

**Table 3:** Bivariate analysis of various clinical and hospital outcomes

	Medication^control^ n (%) 72 (72.7)	Medication^interv^ n (%) 27 (27.3)	Chemical^control^ n (%) 48 (72.7)	Chemical^interv^ n (%) 18 (27.3)
Vital signs^a^				
Normal	63 (87.5)	20 (74.1)	41 (85.4)	16 (88.9)
Abnormal	9 (12.5)	7 (25.9)	7 (14.6)	2 (11.1)
	F-exact, *p* = 0.196		F-exact, *p* = 0.533	
Physical Examination^a^				
Normal	44 (61.1)	18 (66.7)	30 (62.5)	4 (22.2)
Abnormal	28 (38.9)	9 (33.3)	18 (37.5)	14 (77.8)
	*χ*2 = 0.259, *p* = 0.611		*χ*2 = 8.503, *p* = 0.004*	
Diagnostic tests^a^				
Normal	56 (77.8)	17 (63.0)	36 (75.0)	14 (77.8)
Abnormal	16 (22.2)	10 (37.0)	12 (25.0)	4 (22.2)
	*χ*2 = 2.226, *p* = 0.136		F-exact, *p* = 0.545	
Status post ED^a^				
Discharged	59 (81.9)	22 (81.5)	42 (87.5)	15 (83.3)
Admitted	13 (18.1)	5 (18.5)	6 (12.5)	3 (16.7)
	F-exact, *p* = 0.583		F-exact, *p* = 0.467	
	M [IQR]	M [IQR]	M [IQR]	M [IQR]
Length of stay** (hours)	5 [2.7–7.5]	9 [5.4–12.0]	3 [2.0–6.8]	4.5 [2.8–7.2]
	U = 590 (Z = −3.01), *p* = 0.003*		U = 338 (Z = −1.35), *p* = 0.177	
Hospital costs** (USD)	744 [667–861]	813 [667–869]	674 [667–816]	707 [667–849]
	U = 862 (Z = −0.89), *p* = 0.376		U = 399 (Z = −0.50), *p* = 0.619	

*Abbreviation: USD* US dollars, *U* Mann-Whitney test, *Z* Z-score, *F-exact* Fisher exact test, *χ2* Pearson chi-square test, *P p*-value, *M* median, *[IQR]* inter-quartile range [25–75^th^ percentile]

Note: *P-value: statistically significant at <0.05

**Not normally distributed (tested by Kolmogorov-Smirnov, *p* < 0.001)

^a^Categorical variable presented in number (percentage)

Binary logistic and linear regression model were constructed to further investigate the combined effect of all exposures and adjust for all possible confounders, Table 4. The LOS was significantly longer among the group that received a home-initiated non-medical intervention, *p* = 0.035. Toddlers who had lower BMI percentiles tend to be at higher risk for being hospitalized compared to higher BMI toddlers, *p* = 0.018. Any delay in the arrival time to the ED increased the risk of abnormal P.E. by 1.34 times, *p* = 0.003. There were no statistically significant difference between the control and intervention group (in all toddlers) regarding the risk of abnormal V.S., P.E. and Diag tests, *p* = 0.148, *p* = 0.781 and *p* = 0.285 respectively. Also, there were no statistically significant difference between these two groups in terms of in-hospital admission and cost, *p* = 0.499 and *p* = 0.102 respectively.

**Table 4:** Significant clinical and hospital outcomes with their adjusted risk predicators

	Binomial logistic regression				Linear regression	
	Abnormal vital signs	Abnormal physical examination	Abnormal diagnostic test	In-hospital admission	Length of stay (Hours)	Cost (USD)
	Beta (S.E.)	Beta (S.E.)	Beta (S.E.)	Beta (S.E.)	Beta (t)	Beta (t)
	Adj RR (95 % CI)	Adj RR (95 % CI)	Adj RR (95 % CI)	Adj RR (95 % CI)		
	*P*-*value*	*P*-*value*	*P*-*value*	*P*-*value*	*P*-*value*	*P*-*value*
Sex	−0.41 (0.57)	−0.08 (0.44)	0.28 (0.45)	−0.33 (0.55)	0.032 (0.34)	0.05 (0.52)
Female : Male	1:0.66 (0.22–2.03)	1:0.92 (0.39–2.17)	1:1.34 (0.55–3.19)	1:0.72 (0.24–2.11)		
	*p* = *0.471*	*p* = *0.849*	*p* = *0.528*	*p* = *0.546*	*p* = *0.738*	*p* = *0.602*
Age of toddler (Years)	−0.16 (0.75)	−0.39 (0.57)	0.33 (0.53)	0.15 (0.67)	−0.03 (−0.28)	−0.07(−0.73)
	1:0.98 (0.23–4.28)	1:0.68 (0.22–2.07)	1:1.39 (0.49–3.92)	1:1.16 (0.31–4.33)		
	*p* = *0.983*	*p* = *0.494*	*p* = *0.537*	*p* = *0.824*	*p* = *0.782*	*p* = *0.464*
Body Mass Index (Kg/m^2^)	*0.01* (0.18)	−0.09 (0.14)	0.06 (0.12)	−0.51 (0.22)	−0.12 (−1.26)	−0.05(−0.49)
	1:1.01 (0.70–1.44)	1:0.91 (0.69–1.19)	1:1.06 (0.84–1.34)	1:0.59 (0.39–0.92)		
	*p* = *0.966*	*p* = *0.492*	*p* = *0.622*	*p* = 0.018*	*p* = *0.211*	*p* = *0.623*
Substance type	−0.22 (0.64)	0.09 (0.48)	−0.24 (0.49	0.41 (0.60)	0.12 (1.21)	0.13 (1.30)
Drug : Chemical	1:0.81 (0.23–2.82)	1:1.09 (0.43–2.77)	1:0.79 (0.30–2.05)	1:1.49 (0.46–4.89)		
	*p* = *0.736*	*p* = *0.858*	*p* = *0.627*	*p* = *0.502*	*p* = *0.228*	*p* = *0.196*
Witnessed incident	−1.17 (0.64)	−0.57 (0.56)	−0.76 (0.57)	0.48 (0.83)	0.04 (0.48)	0.04 (0.38)
No : Yes	1:0.31 (0.09–1.09)	1:0.57 (0.19–1.69)	1:0.47 (0.15–1.42)	1:1.61 (0.31–8.24)		
	*p* = *0.069*	*p* = *0.309*	*p* = *0.181*	*p* = *0.568*	*p* = *0.630*	*p* = *0.700*
Home management	0.89 (0.61)	0.14 (0.51)	0.52 (0.48)	0.42 (0.62)	0.20 (2.13)	0.16 (1.65)
None : Yes	1:2.43 (0.73–8.08)	1:1.15 (0.43–3.10)	1:1.68 (0.65–4.34)	1:1.52 (0.45–5.08)		
	*p* = *0.148*	*p* = *0.781*	*p* = *0.285*	*p* = *0.499*	*p* = 0.035*	*p* = *0.102*
Arrival time to ED (hours)	−0.21 (0.17)	0.29 (0.09)	0.03 (0.07)	0.10 (0.08)	0.05 (0.49)	*0.02* (0.32)
	1:0.81 (0.58–1.14)	1:1.34 (1.12–1.62)	1:1.03 (0.90–1.18)	1:1.12 (0.95–1.29)		
	*p* = *0.232*	*p* = 0.003*	*p* = *0.625*	*p* = *0.206*	*p* = *0.619*	*p* = *0.751*
Constant	−0.53 (3.22)	1.64 (2.55)	−2.35 (2.22)	5.71 (3.72)	−(1.44)	−(1.39)

*Abbreviation: Beta* coefficient of determination, *SE* standard error, *t* student *t*-test, *Adj* adjusted, *RR* risk ratio, *CI* confidence interval, *Kg* kilogram, *m* meter, *P p*-value, *V.S.* vital signs, *P.E.* physical examination, *Diag* diagnostic tests, *LOS* length of stay, *Cost* hospital bill, *USD* US dollars

Note: *Statistically significant at *p* < 0.05

## Discussion

The general observed findings in this study indicated that the quality of clinical and hospital outcomes were not in favor of the intervention groups (Med and Chem). Having no statistically significant differences between the outcomes of the control and intervention groups rejects the null hypothesis that states that home-initiated non-medical practices improves the toddlers’ clinical and hospital outcomes.

In bivariate analysis, the median of the arrival time to the ED was observed higher in both of the intervention groups compared to their respective controls, Table 3. Any delay in this arrival time significantly increased the risk of abnormal P.E. by 1.34 times, adj. *p* = 0.003 as revealed in the regression model. This indicates that toddlers who underwent a home-initiated non-medical intervention had delays in the time between ingestion and arrival to ED, thus significantly increasing the chance of abnormal P.E. due to this delay. Once more, home interventions not only were proven to be non beneficial in improving the clinical outcomes, but also time consuming, thus putting toddlers at higher risks.

The two main types of home initiated non-medical interventions as reported by parents were orally administered fluids and/or manually induced vomiting. Food and beverages can have a profound impact on many of the medications [23], regardless of whether they are ingested as a prescription or an accident. Effervescent or soluble drugs often contain added sodium which is actually one of the ingredients that aids in better drug absorption, similar to the body’s released bile salts into the gastrointestinal system [24]. Moreover, lemon and other citrus juices can interfere with several kinds of medications [25] by altering the effect of body enzymes that break down (metabolize) drugs in the digestive system and resulting in potentially unwanted side effects [25]. Calcium rich dairy products such as milk and yogurt may compromise the effect of some medications such as antibiotics and thyroid drugs, which decreases their desired effects [26]. In this study, parents decided that such fluids would minimize the effects of the substance ingested without realizing that it all depends on the chemical nature of the substance itself. Findings in this study showed that although some of these fluids might had neutralized the substances’ effect to some extent, the clinical and hospital outcomes showed no improvement statistically even after adjusting for all possible confounders, Table 4.

Manually induced vomiting in this study was a risky and unpleasant practice that exerted physical and psychological stress on toddlers. Similar to other self provoked vomiting behaviors, it is usually associated with a number of unwanted complications such as electrolyte/fluid imbalances and aspirations [27]. Things may become even worse in case the chemical substance ingested was irritating since it may damage the lining of the esophagus, pharynx and oral mucosal surface during vomiting. 

Although there were no published studies with similar study concepts and findings to compare to, the characteristics of the toddlers and poison incidents in this study were comparable to those reported in several studies. The national estimates of incident cases and population based poisoning rates sourced from 100 EDs within the united states announced that in 2004, 72.3 % were toddlers making them indeed the highest risk group among children [28, 29]. This was similar to this study findings as toddlers estimated to 226/315 (71.7 %) in a 2 years period. The Spanish society of pediatric emergencies stated that there is no global difference related to sex among poisoned children [13], which was similar to the findings in this study, *p* = 0.606. Regional studies stated that pesticides and household products [30] as well as paracetamol and other analgesics were the most common ingested substances [30–33]. In our study, antipyretics were indeed the most common (*n* = 25 cases), but the most common chemical was sodium hydroxide products (*n* = 21) followed by petroleum products (*n* = 14). Oral route of poising was the most common in this study 300/315 (95.2 %) which was also similar to literature findings [31].

Abnormal V.S., P.E. and Diag test findings associated with each type of the chemicals ingested in this study, were compared and found similar to the characteristics and symptoms of unintentional poisoning report, issued by a German poison center in 2007 [34]. Hospitalized toddlers were only 16.4 % of this study sample, while the majority received observation, emergency treatments and then discharged home. This was similar to the literature reported in the USA with an admission rate of 13.3% [28]. Toddlers with low BMI were at higher risk for admission in this study, *p* = 0.018, which makes sense as the toxicity level of most poisons such as NSAIDS is measured by the amount of toxin ingested over the toddlers weight [14].

### Limitations

This study has generated outcome analysis on a relatively small sample size. The 2 year limit of the data collection could have been extended further to recruit more eligible cases of poisoning. However, study investigators had to abide with the approved time limit of data collection as per the agreement with the IRB and ED personnel. The data extracted was approached statistically using the more suitable nonparametric tests, yet a larger sample size might have powered the statistical differences between groups. Excluding other age groups, children with positive medical history, and other routes of poison exposure helped in controlling most of the variables that might mask the intervention and increase the chance of outcome assessment bias. Unfortunately, this limited the ability of the study to investigate the outcomes of the home initiated non medical interventions provided to those excluded cases. In addition, the fact that one hospital was involved might limit its ability for generalization to other age groups and settings.

The amount of poison ingested was not accounted for as a potential risk factor due to the diverse nature and form of the substances ingested (powder, cream, fluid, pills, capsules, etc.). Amounts of these diverse substances couldn’t be quantified using a standard measuring unit and it was reported by parents in rough estimates. The fact that the nature of the intervention is based on a reported practice of a parent who was under stress and fear at the time of incident is another concern. Authors suspected a recall and/or a cognitive bias from parents who were reluctant to admit the details of intervention during the initial ED visit. This was overcome by phone contacting the parent at a later time to revalidate the reported practice.

## Conclusions

Home-initiated non medical interventions, whether it’s orally administered fluids and/or manually induced vomiting, didn’t improve the clinical and hospital outcomes among toddlers with orally ingested substances (medications and chemical products). This non-medical practice has lead to a delay in the arrival time to the ED which has put toddlers at higher risk for abnormal P.E. and increased their average LOS. Poisoned toddlers who are at the lower BMI percentiles were significantly at higher risk for being hospitalized.

## Recommendations

Findings in this study were based on a comparative approach between two groups of toddlers with similar health and poison incident characteristics. Based on these findings, it is recommended that parents adhere to the local and international poison management guidelines. Parents need to be informed through community awareness campaigns that the initial response to any suspected or witnessed substance ingestion is notifying a nearby poison center. The launching of a unified hotline poison control number in Saudi Arabia is essential and it’s placement at homes will definitely cut-off delays in the arrival time to ED. Due to the fact that such practices do exist in the community and testing it in randomized control trials is not scientifically and ethically applicable, poison centers need to inquire further on such data from parents who committed such practices. Therefore, it is advisable to incorporate it within the Saudi MOH and DPIC reporting forms for drug over dosage or chemical poisoning, to further investigate the spread and outcomes of such practices.
